# Early fate of exogenous promoters in *E. coli*

**DOI:** 10.1093/nar/gkz1196

**Published:** 2020-01-21

**Authors:** Malikmohamed Yousuf, Ilaria Iuliani, Reshma T Veetil, Aswin Sai Narain Seshasayee, Bianca Sclavi, Marco Cosentino Lagomarsino

**Affiliations:** 1 LBPA, UMR 8113, CNRS, ENS Paris-Saclay, 61 Avenue du President Wilson, 94235 Cachan, France; 2 Current Affiliation: Centre for Clinical Brain Sciences, The University of Edinburgh, Edinburgh EH16 4SB, UK; 3 Current Affiliation: LCQB, UMR 7238, Sorbonne Université, 4 Place Jussieu, 75005 Paris, France; 4 National Centre for Biological Sciences, Tata Institute of Fundamental Research, Bangalore 560065, Karnataka, India; 5 School of Life science, The University of Trans-Disciplinary Health Sciences and Technology (TDU), Bengaluru 560064, Karnataka, India; 6 Sorbonne Université, Campus Pierre and Marie Curie, 4 Place Jussieu, 75005 Paris, France; 7 CNRS, UMR7238, 4 Place Jussieu, 75005 Paris, France; 8 Current Affiliation: IFOM, FIRC Institute of Molecular Oncology, Via Adamello 16, 20143 Milan, Italy; 9 Current Affiliation: Physics Department, University of Milan, and I.N.F.N., Via Celoria 16, 20133 Milan, Italy

## Abstract

Gene gain by horizontal gene transfer is a major pathway of genome innovation in bacteria. The current view posits that acquired genes initially need to be silenced and that a bacterial chromatin protein, H-NS, plays a role in this silencing. However, we lack direct observation of the early fate of a horizontally transferred gene to prove this theory. We combine sequencing, flow cytometry and sorting, followed by microscopy to monitor gene expression and its variability after large-scale random insertions of a reporter gene in a population of *Escherichia coli* bacteria. We find that inserted promoters have a wide range of gene-expression variability related to their location. We find that high-expression clones carry insertions that are not correlated with H-NS binding. Conversely, binding of H-NS correlates with silencing. Finally, while most promoters show a common level of extrinsic noise, some insertions show higher noise levels. Analysis of these high-noise clones supports a scenario of switching due to transcriptional interference from divergent ribosomal promoters. Altogether, our findings point to evolutionary pathways where newly-acquired genes are not necessarily silenced, but may immediately explore a wide range of expression levels to probe the optimal ones.

## INTRODUCTION

The high fraction of mobile genes in bacterial genomes is a source of a great diversity of phenotypes. This large diversity challenges the very concept of species, and has enormous importance for understanding pathogenicity and antibiotic resistance (1). At the genetic level, *Escherichia coli* genomes vary dramatically in their sizes ranging from 4.5 to 6 Mb. Comparative genomic surveys of *E. coli* have shown that there is a core set of genes which is highly conserved across the species and coexists with a large pangenome, the set of genes that can be gained by horizontal gene acquisition from other species (2). Indeed, bacteria acquire exogenous DNA by transformation (of naked DNA from the environment), transduction (of DNA from bacteriophages) or conjugation (from fellow bacteria through molecular pipes such as pili) (3). In order to be functional, exogenous acquired genes often need for the metabolic and the regulatory circuitry of the cell to be rewired (4,5). Furthermore, expression of a foreign gene can interfere with the resources allocated for endogenous gene expression. Therefore, horizontally acquired genes must be regulated (6).

A primary mode by which the expression of horizontally-acquired genes is regulated is believed to be transcriptional repression, which is achieved by proteins such as H-NS in enterobacteria, including *E. coli* ((7–10), reviewed in (11)). Many previous studies support both the need of initial repression of acquired genes, and the view that H-NS repression is relevant for the successful establishment of these genes (5,6,12,13). H-NS is among several ‘global’ transcriptional regulators that affect the expression of hundreds of genes in *E. coli*. It binds to AT-rich or intrinsically bent DNA sequences and forms structures such as stiff rods or DNA–protein–DNA bridges, which might act as geometrical motifs for transcriptional silencing (14,15). Many H-NS binding regions are up to a few kilobases long. The length of these binding regions correlates with the degree of transcriptional repression imposed on the target gene (9,16) and genes regulated by H-NS are very highly expressed in the absence of this repressive control (17,18). Since the levels and activity of H-NS depend on environmental conditions and growth rate, this level of regulation allows for a coordinated gene-expression change needed for cellular adaptation. Studies of gene expression of inserted reporter cassettes at different genomic locations (16,19) have demonstrated that gene expression of an identical regulatory system can vary greatly, beyond the effects of gene dosage, for three main reasons, supercoiling, activity of neighbour promoters and H-NS regulatory activity. A gene’s local environment can thus provide a fitness advantage, associated to the selection of the gene’s position over evolution.

Additionally, at least through the action of H-NS, which is a notorious nucleoid-shaping protein (20), the dynamics of horizontal transfers is related to the physical organization of the chromosome. An important question to be addressed is whether and how the organizational features of the *E. coli* chromosome -such as the ‘macrodomain’ architecture (21–24)- are correlated with gene acquisition and control of gene expression, particularly of acquired genes (25).

Horizontally transferred genes are often clustered along the genome (3,23,26–28). In part, this reflects joint transfer of functionally co-dependent genes that would provide no benefit if transferred independently. In part, however, this reflects the existence of ‘permissive’ zones along the chromosome, which experience recurrent integration and high turnover. Permissive zones can originate or be reinforced through physical integration biases, where the presence of integrases and/or recombinogenic sites facilitates acquisition of genetic material (1). Additionally, in many species including *E. coli*, horizontally acquired genes preferentially accumulate near the (AT-rich) terminus region (1,2,23), possibly to avoid deleteriously high expression near the origin due to gene copy number effects.

The genome sequence organization of a given species is a result of selection pressure and architectural constraints. Some of these have been clearly identified (3,6,17,28,29). For example, highly expressed, newly acquired genes must be kept from interfering with the expression of essential genes. However, comparatively little is known about the early dynamics of acquired genes. Do clear physical insertion biases emerge? What are the phenotypic impacts of inserted genes and how are they linked with expression levels? How, in turn, is gene expression a consequence of the locus of insertion? Are they immediately silenced and do H-NS and nucleoid organization play a role?

**Figure 1.**
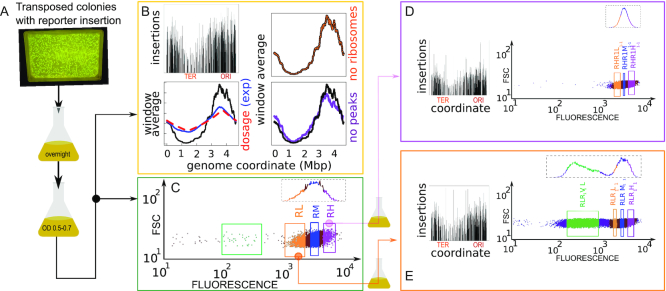
Insertion localization and sorting by gene expression. (**A**) Experimental pipeline. Massive transposon insertion of a GFP reporter gene cassette in ∼100 000 founder strains was tested by plating on kanamycin-selective agar and PCR. Surviving colonies were mixed, grown overnight in LB, resuspended and grown to a fixed OD. (**B**) Sequencing of resulting parental populations yields the locations of the insertions, shown in the top-left panel (y axis are counts in logarithmic scale). The bottom panel compares a 3 kb sliding average of the coverage (black line, y-axis rescaled for comparison) with the prediction from gene dosage, and the experimental dosage (red dashed line) measured by whole-genome sequencing (blue line) the right panels are controls that the trend of insertions copy number is not due to ribosomal genes (orange line) and to the insertions with top 10% coverage (>3000 reads/bin, purple line). (C–E) Forward scatter versus GFP expression measured by flow-cytometry. FACS Sorting by the level of fluorescence was performed on a total of four rounds (see Supplementary Figure S1). Selecting for high expression (RH) from the parental population (**C**) yielded a population with a similar distribution of gene expression (**D**), while selecting for low expression yielded a population with a bimodal distribution of gene expression (**E**). Insets in panels (D) and (E) show insertions found by population sequencing (y-axis are counts in logarithmic scale), with overall similarity but local differences.

To access some of the above questions, we devised an experimental assay (Figure 1) where a cassette including an antibiotic resistance gene and a GFP reporter under the control of a highly expressed ribosomal promoter is inserted systematically in the genome, and the resulting mixed and clonal populations are analysed by sequencing and single-cell biology methods. This methodology allows us to describe statistical tendencies for a reference promoter to be inserted and initially maintained in specific chromosomal contexts, as well as to characterize its fate in terms of both gene expression activity and noise of the transcription reporter constructs at different insertion sites on the genome.

## MATERIALS AND METHODS

Detailed Methods are available as Supplementary Materials.

## RESULTS

### Efficient protocol for production and characterization of systematic exogenous reporter insertions

The promoter chosen to control GFP expression in the randomly inserted cassette is the rrnBP1 promoter of the rrnB ribosomal operon. We chose this well-characterized highly expressed promoter because it is regulated by changes in DNA supercoiling and by the abundant nucleoid proteins Fis and H-NS (30). We also considered, as a control, a shortened version of the promoter lacking regulation by the nucleoid proteins Fis and H-NS but still regulated by DNA topology.

Figure 1A describes our pipeline (see Methods and Supplementary Figure S1). On the order of 10^5^ transposed colonies (this estimate was based on manual counting as we knew the number of transposed colonies in each plate) were mixed and grown overnight in minimal medium. This population was regrown to a fixed OD in the same medium, and initially assayed by population sequencing (Figure 1B and flow cytometry Figure 1C–E). We then used a cell sorter to select sub-populations based on gene expression levels (Figure 1C–E). Each of these subpopulations was grown overnight, regrown to exponential phase and then sorted again as a function of GFP content, for a total of four rounds (Supplementary Figure S1). Finally, 658 randomly hand-picked clonal populations were individually characterized by flow cytometry. A subset of 96 from the 658 clonal populations representing different sorted populations were randomly selected and sequenced, and 90 of these were used to measure gene expression and growth rate in a plate-reader assay (see Supplementary File SF1). A smaller selected subset of clones was used to measure the dynamics of gene expression in single-cell microcolony growth assays by epifluorescence microscopy.

### Bimodal distribution of gene expression in parental populations and low-expression sub-populations

Comparison of the fluorescence distribution in the sorted populations obtained from the high- (RH) and low-expressing (RL) fractions of the parental population (Fig 1C–E) shows that the low-expressing (RL) population have a sub-population of clones with very low expression. In the parental population (Fig 1C), some of these low-expression clones are already visible (green box). Sorting them from the RL population gave rise to the population RLR1V1L.

Outside of this low-expression peak, the distribution of gene expression has little variation in the sorted populations compared to the parental one. This variability is the combination of the variability of promoter expression across single cells that are clonal (i.e. where the insertion is in the same exact position) and the variability of mean expression between clones with different insertion locations. Thus, the clonal variability should be considerably high in order to account for the fact that the overall pattern of variability is robust in the sorted sub-populations (which contain less clonal variants). There are, however, some important differences in the distributions, mirrored by differences in the location and frequency of the insertions in the sorted populations, which turn out to be significant (see below).

### Insertions are non-uniform and sparse and are more biased towards the replication origin than justified by gene dosage

TraDIS Sequencing of FACS-sorted populations based on GFP expression shows the presence of transposon insertions at different chromosomal positions. Coverage of the insertions is uneven and sparse (Figure 1B, D, E). In addition, there is a bias with respect to genome coordinate, with a higher insertion frequency close to the replication origin and a lower insertion frequency close to the terminus. The distributions of insertion frequencies in the parental populations of the P1-short and P1-long promoter insertions showed the same qualitative features as a function of genome coordinate (Supplementary Figure S2). Populations derived from the high or medium GFP expression populations show a bias for insertions closer to the origin of replication (Supplementary Figure S2). We also noted that populations derived from the low GFP expression populations, particularly those filtered for very low expression levels, showed high-frequency insertions in ribosomal regions (Supplementary Figure S2).

We tested a possible role of gene dosage in the origin-to-terminus bias of insertion frequency. The samples are in early log phase in LB medium at 37°C when they are exposed to the transposon. There is therefore a higher number of copies of the chromosome close to the origin than to the terminus. Estimating the dosage from the Cooper–Helmstetter model (34), and assuming an insertion rate proportional to the dosage, we computed the expected insertion bias, keeping into account the population age-structure (see SI text).

Figure 1B shows that the dosage estimated theoretically agrees very well with whole-genome sequencing of genome copy number, but is not sufficient to explain the stronger origin-terminus bias of the insertions. We also verified that this bias was not due to the insertions with top 10% coverage and to the insertions on ribosomal genes. The additional bias may be due to additional factors such as DNA supercoiling or biased binding of nucleoid proteins and differences in nucleoid compaction (35–38). Additionally, the density of insertions shows a slight left-right asymmetry with respect to the origin, which is visible when the sliding average of insertions is compared with the prediction from dosage (Figure 1B). We verified that a model with time-dependent insertion rate, i.e. where the insertion rate *r* increases with time *t*, *r*(*x*, *t*), can fit the data, using insertion rate growing as a power law in time (see SI text). However, there is no empirical motivation to assume such cooperative behaviour in insertions that occur *in different cells*. Alternatively, since the exponent linking expected dosage and measured insertions is close to three, one can also hypothesize a cooperative effect of technical or biological origin, but we could not produce a technical or biological explanation for such a simple cooperativity.

### H-NS binding sites are enriched at insertions positions

In order to better characterize the genomic positions of the insertions, we investigated the statistical tendencies for localization of insertions using the gene lists from the NuST database (33). This database contains a large panel of published gene sets measuring several genomic properties such as binding of nucleoid-associated proteins, including several H-NS data sets (see Supplementary Table S1 for a detailed description of each data set). To score for significance, we compared the co-occurrences of insertions and genes with 5000 realizations of a shuffling null model (see Supplementary Methods for details). Note that the null model subtracts the empirical sliding average of insertions, and not the dosage, thus the results are net of the overall enrichment around the origin. The analysis was applied to the population-sequencing data for the insertion sites in both the parental populations as well as in the ones that were sorted for gene-expression levels.

**Figure 2.**
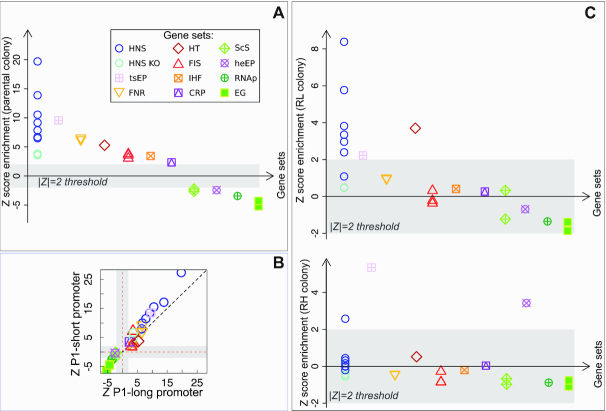
Enrichment of insertions for H-NS and other global regulators. (**A**) *Z*-score of enrichment tests for different gene lists (see Supplementary Table S1 for a full legend). H-NS binding sites (from ChIP-seq and ChIp-ChIP data) and H-NS perturbations experiments (from (39)) are highly enriched (circles), indicating a strong positive association of insertions to H-NS binding regions starting from the parental colony. Other global nucleoid regulators (FNR, Fis, IHF, CRP, see legend), and a list of horizontal transfer genes (HT, see legend) also show positive association, lists of essential genes (filled squares) show strong negative enrichment. (**B**) Comparison of the two different promoter tested (with and without Fis and H-NS binding sites) shows a similar behaviour. (**C**) Comparison of parental and sorted populations (see Figure 1C–E) shows that H-NS association maintains a strong significance in the low-expression population, and loses significance in the high-expression population, where FNR sites remain highly enriched.

This analysis, summarized in Figure 2A, shows that H-NS binding is the main property associated with any insertions (even before any sorting by gene expression is performed). The light blue circles in this figure refer to different genome-wide H-NS occupancy (ChIP-ChIP and ChIP-seq) data sets, obtained in different conditions (see Supplementary Table S1). The dark blue circle refers to genes that are sensitive to H-NS knockout under perturbations that make supercoiling more positive (39). The crossed square refers to generic transcriptionally silenced extended protein occupancy domains, which are largely made of H-NS bound regions. All these sets are correlated but not identical, and essentially contain different categories of H-NS bound regions.

Importantly, a strong enrichment is shared with putative horizontal transfers detected from sequence properties (among which AT-richness (40), red diamond in Figure 2A). Indeed, the full list of insertion sites is enriched in H-NS target genes regardless of the expression level (Supplementary Table S2). We also found an enrichment on H-NS binding sites in the surroundings (10 kb regions) of the insertions compared to random sites (Supplementary Figure S2).

The positive local association of H-NS binding sites with insertion sites is in agreement with a common preference for AT-rich regions. As previously mentioned, genomic insertions generally have a reported bias for AT-rich regions (1,3,25), and AT-rich regions are also the preferred binding targets for H-NS (9,10,20,23). We looked for a correlation between all insertions (regardless of their association with H-NS) and AT-rich regions. In order to do this, we compared the distribution of AT-bias in the sequences surrounding insertions with a random sample of the background sequences of the genome. A one-tailed Kolmogorov–Smirnov test (*P*-value <10^−16^) suggests that there is a significant difference between the two distributions, with the sequences surrounding the insertions being richer in AT than the background ones (Supplementary Figure S2). The role of H-NS has been proposed to inhibiting insertions, in addition to repressing events of spurious transcription (12,18,41,42). Our results lead us to conclude that, in the tested conditions, at these fast growth rates, H-NS does not appear to inhibit physical events of transposon insertion efficiently. The correlation analysis performed here does not allow us to conclude that AT-richness causes the enrichment of both insertions and H-NS binding. Another causal chain is possible, but appears less likely, where H-NS binding facilitates insertions, thereby driving them towards AT-rich regions. We also note that the Tn5 transposon has been reported to be biased towards *GC-rich* regions (43), by a similar analysis than that performed in Supplementary Figure S2, but in different conditions, and in a different organism. This previous result makes the positive association that we find between insertions and AT-rich regions more intriguing. Additionally, Figure 2B shows that the significant enrichments of the different gene sets for insertions are consistent across the two promoters used here (P1-short and P1-long) used here (see also Supplementary Table S5), as expected from a lack of a role of the donor sequence on insertion bias.

### Other global regulators are enriched at insertions positions

We now proceed to discuss other gene sets that share enrichment for any insertions (visible in Fig 2A). Of notable significance are targets of global regulators Fis (which alters the nucleoid state to aid transcription in exponential growth) and FNR (which alters the distribution of RNA polymerase in response to oxygen starvation). This could be related to AT-richness bias of the binding site of these proteins or to high transcriptional activity (and thus accessibility for insertions) of these genes (41,42). It is reasonable to expect that Fis targets are more active in LB medium. However, we found that transcriptionally active RNAP binding regions (measured by ChIP-chip during rapid growth (44)) are *under-represented* for insertions, which suggests a negative interaction between RNAP binding or transcriptional activity and insertion frequency (Supplementary Table S3). Finally, a milder but significant over-representation for insertions was found for CRP and IHF targets, genes that are sensitive to supercoiling perturbations in an H-NS knockout background, and genes with trans-membrane domains (Supplementary Table S4).

Conversely, essential genes (filled green squares in Figure 2A) are the most under-represented set for insertions, as expected (Supplementary Table S3). The other under-represented gene sets for insertions comprise RNAP targets in rapid growth (crossed dark-green circle in Figure 2A), genes whose promoters are sensitive to supercoiling changes and highly transcribed occupancy domains, suggesting a negative correlation between insertion probability and transcriptional activity.

### H-NS binding is the sole over-represented signal in low-expression clonal populations

Finally, we compare the parental population with the ones sorted for gene expression levels (Figure 2C and Supplementary Table S6). The comparison of the insertion sites of the high and low expressing populations from the P1-long promoter strains shows that the low expressing population is found preferentially within H-NS binding regions, while the high expressing population is not. Indeed, the low-expression (RL) population maintains a similar association as the parental population with H-NS, and no other binding protein. Conversely, the RH (high expression) populations show no association with H-NS, and only maintain some enrichment with FNR (Figure 2C and Supplementary Table S6). Hence, despite the lack of an effect in inhibiting transposon insertion, H-NS does appear to regulate the level of gene expression of the inserted sequences.

Overall, these results point to a more complex role than is expected for H-NS in modulating genome accessibility and gene expression of recently acquired genes.

### Flow-cytometry analysis of clonal populations shows variable noise and gene-expression properties

Each of the sorted populations was plated separately to yield individual isogenic (clonal) colonies. To gain further insight into these differences in gene expression, 658 individual clones were hand-picked from the different populations and grown in 96-well plates to measure the average fluorescence and its standard deviation by flow cytometry. From these, 90 clones where chosen to measure both the fluorescence and the growth rate in a plate reader in different growth media.

This analysis yields the following main results (see Supplementary Figures S3–S5)

**Figure 3.**
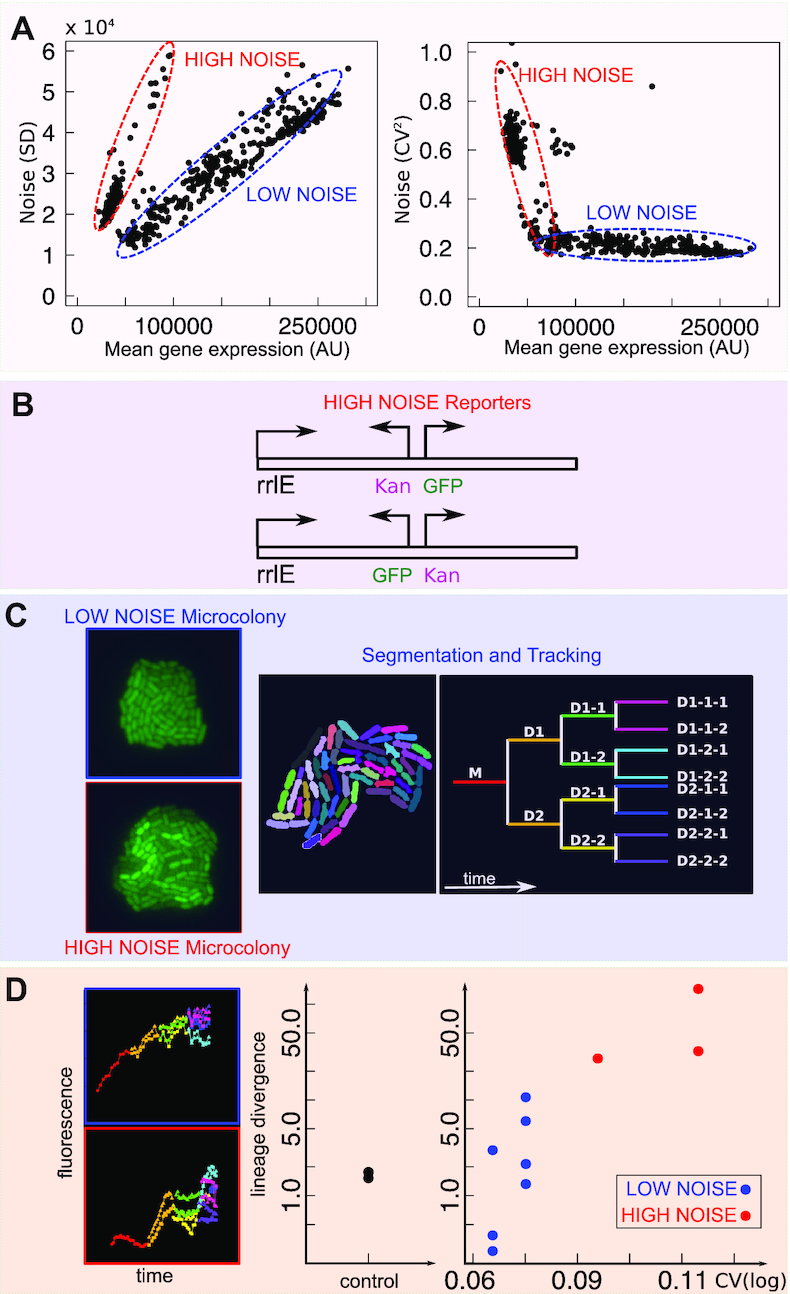
Noisy promoters emerge from transcriptional interference within the rrlE ribosomal operon. (**A**) Characterization of noise of 658 clonal populations from flow cytometry. Left: Standard deviation vs mean GFP expression. Right: Noise (CV^2^) versus mean GFP expression. (**B**) All the tested ‘noisy’ clones by sequencing typically show association with the *rrlE* ribosomal RNA operon (see Supplementary File SF1). (**C**) Characterization of promoter noise by microcolony time-lapse growth assay. Microcolonies were grown, imaged, segmented and tracked for three generations. (**D**) An example of the resulting lineage-specific gene expression data is shown on the left. The plot on the right quantifies the lineage divergence of gene expression (time average of the absolute gene expression difference) of the different clones, comparing it with the CV measured from flow cytometry (see text). High-noise clones show a large lineage divergence - indicating a possible switching behavior.

-There is agreement between a clone’s level of fluorescence and the average level of fluorescence of its original population (Supplementary Figure S3), as measured by both flow cytometry and the fluorimeter. Specifically, the clones from the low-expressing populations have a significantly lower average level of fluorescence.
-The magnitude of the difference between high- and low-expressing clones depends on the growth medium. The difference is greater in the faster growth medium (Supplementary Figure S4).
-The very low-expression strains (Figure 1E) show low expression regardless of the growth media, except for a few outliers showing out-of-trend expression (higher than expected) in the poorer medium (Supplementary Figure S4). The last two observations are consistent with the known growth-rate dependence of H-NS activity and ribosomal operons transcription rate (16,45,46). This result shows that different regions of the genome can have different growth-rate dependent properties.
-Each clonal population (with a single insertion site, as verified by sequencing) shows a distribution of fluorescence whose spread does not depend solely on the average expression level (Figure 3A and Supplementary Figure S5).

### A set of ‘noisy’ insertion sites

We found that in all the clones the standard deviation of gene expression is proportional to the mean, meaning that most of the noise shown by the promoter is extrinsic, regardless of expression level and insertion location (Figure 3A). This is expected from the high level of expression of the rrnBP1 promoter (47). However, a subset of clones show a higher level of noise where the scaling of the standard deviation with the mean follows a steeper slope. In other words, these clones show much larger gene expression variations than expected. All of these high-noise clones were very-low expression bacteria obtained from the RL (low-expression filtered) population. Hence, these clones likely explain the very-low expression sub-population (in green in Figure 1E) in the distribution of gene expression of the RL (low-expression filtered) population. When tested for their distribution in single-cell gene expression by flow cytometry, these low-expression high-noise clones did not show bimodal distributions. Rather, they showed disperse and skewed distributions, whose range overlaps with the expression level of other low-expression clones (Supplementary Figure S5A, B). Supplementary Figure S5C–G recapitulates the noise properties of all the picked clones from different rounds of selection. It is clear that high-noise clones become more frequent in successive sorting rounds where low or very-low expression cells are selected. The following two sections deal with a more detailed characterization of the properties of these low-expression, high-noise insertions.

### Noisy sites are associated with the insertions within ribosomal operons

To characterize the set of clonal colonies from different populations, we performed whole-genome sequencing on 90 selected clones. The locations of all the insertions of these clones are listed in Supplementary File SF1. Thirty two clones out of the 90 selected samples show the presence of insertion within a rRNA operon from whole-genome sequencing data. These clones were very-low expressing clones derived from RL filtered populations. For example, in the clones from the RLR1V1L sorted populations we tested a total of 16 insertions, of which nine were in rRNA regions (Supplementary File SF1). In order to verify these short-read assignments of insertions sites, long-read nanopore sequencing was used on five of these 32 clonal samples. This analysis confirmed the presence of the transposon insertion within the 23S ribosomal RNA (rrlE) of the rrnE operon. To recapitulate these results, Supplementary Figure S6A shows the association of clones with high-noise promoters with rrlE insertions. Some of the non-rrlE high-noise clones revealed the presence of multiple (up to three) insertion sites, which can explain the large variance in gene expression of these clones (see column D of Supplementary File SF1 for a list of the multiple insertions and their coordinates). Indeed, we found that normalization of gene expression by the copy number of the promoter removed these outliers (Supplementary Figure S6B, C). Hence, we believe that the true high-noise phenotype should almost exclusively be associated with rrlE insertions.

Insertion within ribosomal operons is tolerated because *E. coli* has seven copies of ribosomal operons. To discover the orientation of the cloned sequences with respect to the rrlE promoter position, we used the blast results of *de novo* assembled contigs with the flanks of cloned insert sequence and we compared them with the reference genome. Most of the insertions showed an opposite orientation of the inserted promoter with respect to the rrlE promoter sequence. Separately, we checked the orientation of the inserted GFP cassette in few selected Illumina samples for which the blast results showed reasonable overlap with the genome locus. This confirmed the opposite orientation of GFP with respect to the rrlE promoter sequence in most samples. Strong promoter competition may explain the very low levels of GFP transcript production by which these clones were isolated, as well as the high variability.

We found that the trends of gene expression with growth rate were consistent with the hypothesis of competition with a strong promoter: while most of the other clones increase their expression with growth rate (in agreement with the known regulation of the rrnBP1 ribosomal promoter) the noisy clones decrease in expression with increasing growth rate, in agreement with the idea that their expression is repressed by an interference with transcription of the increasingly transcribed ribosomal operon (Supplementary Figures S3 and S4). Additionally, the mild reduction in growth rate for insertions giving different mean GFP expression suggests that the cost associated to GFP expression and the possible interference with the ribosomal operon are not dominant for these insertions (Supplementary Figures S3 and S4).

### Noisy promoters may perform switching

The previous analyses strongly indicate an association of the high-noise insertions with an interference of the insertions with ribosomal operons. To gain more insight on the temporal dynamics of these high-noise inserted promoters, we measured gene expression noise in time-lapse microscopy data on growing microcolonies (Figure 3C, D). We compared the change in GFP gene expression over time of single cells from clones carrying noisy and non-noisy promoters for three to four generations and quantified the differences between gene expression in different lineages. The divergence between lineages was quantified as the time average of the absolute value of the gene expression difference between sister cells.

Bacteria were grown on an agar pad to form a microcolony. The time-lapse data in the formation of the microcolony was segmented to obtain the change in the average cell fluorescence as a function of time Figure 3C. An example of gene expression of two lineages, one from a noisy clone, as measured by flow cytometry, and one from a control clone (where the cassette was inserted specifically between two converging genes, AidB and yjfN) is shown in the left panel of Figure 3D. The right panel of Figure 3D quantifies the divergence of gene expression along lineages for different clonal microcolonies, corresponding to clones where the promoter is inserted in different positions. Figure 3D shows that in microcolonies from high-noise clones different lineages emanating from the same single cell tend to diverge more in gene expression as time progresses than in the control or low-noise clones. This result points to the possible presence of switching behaviour in the high-noise clones.

## DISCUSSION

Our results directly show that the probability of DNA insertion in the *E. coli* genome by a transposon is biased, before any long-term selection may act, other than that related to overnight growth. First, there is a stronger origin-to-terminus bias than explained by gene dosage imbalance, second, insertion probability is higher in AT-rich regions of the genome. This may seem surprising, because these are regions that are preferentially bound by the H-NS protein, which has been proposed to act as a barrier to horizontal gene transfer (3,6,8,10,28,48). However, physical components such as differences in DNA supercoiling (35,49,50) and the biophysical properties of AT-rich DNA (lower melting barriers, different stacking energy, etc.) may play a role in establishing these biases. In particular, the measured origin-to-terminus gradients of supercoling (51) and gyrase binding (52) are compatible with the hypothesis that the probability of insertion is increased in regions with higher negative supercoiling, which may explain the origin-to-terminus positive bias for insertions (not justified by origin-to-terminus differences in gene dosage). Note that this insertion bias (only transposon type) should not be confused to the transcriptional consequences of supercoling on the donor promoter. A different promoter sequence is not expected to affect the insertion probability. On the other hand, changes in gene expression levels depend on promoter sensitivity to supercoiling and local context.

An important technical point to address is the role of kanamycin selection in these experiments. If the donor sequence including the kanR cassette is inserted in a locus where it is completely silenced by H-NS, one might not be able to see the insertion. However, our data show that selection itself does not preclude the identification of an insertion site, since insertions are not excluded from H-NS occupancy-rich regions, as it would be expected if complete silencing of KanR expression had taken place. We do observe that promoters inserted in H-NS rich regions are on average expressed less than the others. We also note that we carried out the transposon reaction in mid exponential phase cells growing in a rich medium, LB. In these conditions the concentration of H-NS is lower due to a high dilution rate (16). However, at the faster growth rates, H-NS is known to still play a role in the repression of ribosomal promoters by binding to higher affinity sites (37,53). Our results suggest that there is probably not enough protein to also cover the lower affinity (nonspecific) binding to AT-rich regions, in order to inhibit transposon insertion (11). This is in contrast to a previous study in *Vibrio cholerae* that has shown that only in the absence of H-NS there was a higher probability of insertion in AT-rich regions of the genome (12). These results lead us to further suggest that the role of H-NS in regulating the probability of genomic insertion of horizontally acquired genes may depend on the growth conditions and on the specific strain.

Our results also indicate that the dilution of H-NS in rapidly growing cells does not prevent the establishment of a low-expressing population biased for the insertions associated with H-NS binding. Hence, they are consistent with the role of H-NS as a silencer of newly acquired genes. Indeed, we observe that once the full length rrnBP1 promoter cassette is inserted in the genome, its level of expression is lower if it is found near H-NS rich regions. This cassette includes a higher affinity H-NS binding site within the full length rrnBP1 promoter and an AT-rich *gfpmut2* gene sequence stabilizing the formation of H-NS dependent repressing complex. The shorter version of the promoter (P1-short), lacking the high affinity H-NS binding site, does not appear show a stable sub-population of very-low fluorescence clones (data not shown), showing that the high affinity site of the promoter is important in nucleating the repressing oligomeric structure, a question that we are still exploring. In summary, the level of expression of the clones can thus be very heterogeneous, depending on local properties of the site of insertion and the sequence of the fragment.

The cell-to-cell variability of gene expression within a given isogenic clone can vary significantly, but it typically scales with the mean level of expression as expected from extrinsic noise (45,47,54). This is expected from the promoter used here, rrnBP1, which is a strong promoter, resulting in a high level of expression. The change in the CV as a function of mean expression therefore remains for the most part relatively flat, corresponding to the extrinsic noise regime.

However, in some of the very low expression clones the noise varies in a way that is not expected from the known pattern of gene expression noise correlations that have been described previously (45,47). We therefore characterized those clones that have a higher level of gene expression noise and found that in these cases the insertion has taken place within a ribosomal operon. *Escherichia coli* has seven copies of ribosomal operons, therefore insertion inside one of them does not have a high cost and is not selected against, at least in the short term of this experiment. This results in interference between two transcription processes driven by very similar promoters, of similar strength. Furthermore, the initiation frequency of ribosomal promoters is high enough at fast growth rates that most of the time the operon sequence can be assumed to be covered by transcribing RNA polymerase ‘trains’ (55). This creates a block for RNA polymerase to bind to the promoter that is found within the operon, creating a stable ‘off state’. However, from time to time RNA polymerase manages to bind to the newly inserted promoter, perhaps after the DNA replication forks have erased the memory from the competing process, starting its own ‘train’ of GFP production. Such transcriptional interference is a well-known phenomenon (56). Our result on promoter noise also suggests that in the tightly packed bacterial genomes, transcription interference with newly inserted genes might be a natural source of innovations in terms of gene expression noise on evolutionary time scales, as previously speculated for eukaryotes (57).

Altogether, the main novelty of our study is the support for a high (initial) tolerance for insertions with a wide range of expression levels, which challenges the standard view that H-NS not only silences horizontally acquired genes (because they are more AT-rich) but also inhibits insertion in AT-rich regions of the genome. We find that in the tested growth conditions, H-NS does not inhibit insertions in AT-rich regions. However, it can still decrease expression after the insertion has taken place. These findings support the following evolutionary scenario. When a novel gene enters the genome, it is more likely found in a region that is controlled by H-NS, for reasons that most likely have nothing to do with fitness, but have to do with the physico-chemical properties of the DNA in AT-rich regions. However, the wide range of expression levels that we find show that the gene is not necessarily immediately silenced. Rather, the different insertion positions allow it to sample a wide range of expression levels (including silencing), at (initially) equal promoter strength, while interacting from the start with the cell’s housekeeping physiology. We believe that this inherent bet-hedging and exploratory stage may be a key ingredient of genome plasticity, and is underestimated in our current narrative of the process of horizontal transfer, which is centered on the average outcome, and establishes a strict time hierarchy between stages where an exogenous gene is first silenced and then reactivated.

## DATA AVAILABILITY

The sequencing data are available on the Sequence Read Archive (SRA) (https://www.ncbi.nlm.nih.gov/sra) under BIoproject accession IDs numbers PRJNA575574 (WGS Data) and PRJNA575567 (TRADIS data). The (processed) cloned insert FASTA file is available as Supplementary File SF2.

## Supplementary Material

gkz1196_Supplemental_Files
