# Supplementary material for: Early fate of exogenous promoters in E. coli
Source: Nucleic Acids Res. 2020 Jan 21;48(5):2348–56. doi: 10.1093/nar/gkz1196 (PMC7049719; doi:10.1093/nar/gkz1196)
Supplement: gkz1196_Supplemental_Files [file gkz1196_supplemental_files.zip › Cefipra_Paper_SI_wmethods.pdf]

Supplementary materials for Yousuf *et al.*

"Early fate of exogenous promoters in *E. coli*"

Materials and Methods

Experimental procedures

Generation of the transposon library.

The transposon used for genome wide insertion is derived from Ez-Tn5^{TM} custom transposon kits (Epicentre). The insert is a cassette composed of the resistance gene to kanamycin and the *gfpmut2* gene under control of the well characterized ribosomal promoter rrnBP1. The rrnBP1 promoter is sensitive to both changes in DNA supercoiling and to regulation by the small metabolite ppGpp. The full length rrnBP1 promoter, "P1-long", includes binding sites for H-NS and Fis. We also used as a control a shorter version "P1-short", which lacks the Fis sites and has reduced H-NS binding (1).

The level of expression of GFP under control of rrnBP1 can be thus used to assess the effect of nucleoid associated proteins such as H-NS.

The cassette is cloned between the BamH1 and Sal1 sites of the Ez-Tn5^{TM} pMOD^{TM}-3 construction vector. The 5' phosphorylated primers used for amplifying the insert with mosaic ends of the transposon are the following,
5'-CTGTCTCTTATACACATCTCAACCATCA-3' 5'-CTGTCTCTTATACACATCTCAACCCTGA-3'

200 nanograms of the purified PCR amplified fragments were mixed with the transposase enzyme and glycerol at 37°C for 1 hour. Bacteria were grown to early log phase in LB at 37°C before the transposons were added. The transposon mixture (1μl) was electroporated into the wild type strain (BW25113). After electrotransfromation, the bacterial cells were allowed to recover in SOC (rich) medium at 37°C for 2 hours without antibiotics. The cells were plated on kanamycin (50μg/ml) plates, and grown overnight at 37 degrees to select the transformants. Genomic DNA was isolated from the recombinant clones and internal primers binding to the transposon-specific regions were used to verify the insertion by PCR. A typical electroporation experiment yielded 15,000 to 20,000 transformants. In total, approximately 10^5 transformants were pooled from 10 identical electroporation experiments. These are called the parental clones or the parental population in the main text.

Sorting of transformed clones using FACS.

The transformed cells were pooled together from the Kanamycin agar plate and were grown overnight at 37°C in minimal medium (0.5% Glucose + 0.2% CAA). On the following day, 1% of the primary culture was diluted to 1:500 and grown in a secondary culture in minimal medium until the OD

reached 0.5 to 0.7 (mid exponential phase). Different populations were sorted by FACS (Fluorescent Activated Cell Sorting) by the level of GFP expression. The populations in the left and right tail of the distribution (roughly below and above 0.7 standard deviations from the mean) after the first round of FACS, and those around the mean of the distribution (roughly 0.5 standard deviations from the mean) were sorted and labelled (see Fig.1 and Supplementary Fig. S1 for a graphical scheme) as low (RL), medium (RM) and high (RH) expression respectively (R stand for Rounds of FACS). Subsequently, the RL sorted population was regrown overnight at 37°C diluted to 1:500 the next day and grown in a secondary culture in minimal medium until the OD reached 0.5 to 0.7. They then underwent a next round of FACS to yield 4 sorted subpopulations,

RLR1V1L1 (very low), RLR1L1 (low), RLR1M1 (medium) and RLR1H1 (high) whilst the RH yielded three populations, RHR1L1-1 (low), RHR1M1-1 (medium), RHR1H1-1 (high). A third round of regrowth and FACS was performed on RLR1V1L1 (very low), RLR1L1 (low), and RLR1H1 (high) selected from the low expressing population, and on two populations, RHR1L1-1 (low) and RHR1H1-1 (high) from the high expressing RH population. Only the very low expressing population, R1L1R2V2L2 from the third round of FACS underwent a fourth round of regrowth and FACS resulting in four subpopulations that were sorted depending on their levels of expression. All the populations from the four rounds of FACS were stored at -80° in glycerol.

Library preparation and sequencing of the different populations.

Tradis sequencing for FACS sorted populations. Bacterial populations sorted based on GFP fluorescence from FACS experiments were grown in LB media containing the antibiotic Kanamycin for 16 hours at 37°C at 200 rpm. The genomic DNA samples were isolated from these populations using the QIAGEN DNA purification kit protocol. Genomic DNA was sonicated (Covaris S220) to 350 bp fragments using standard factory settings, was end-repaired using Truseq Nano DNA LT End Repair and A-Tailing mix. The end-repaired samples were ligated using Truseq Nano DNA LT ligase enzyme to adapters described by ref. (2). Adapter-ligated fragments were purified and PCR was performed to enrich the fragments. The PCR primers were designed according to ref. (2). For the PCR, we used Truseq Nano DNA LT PCR mix with thermal cycler conditions of 95°C for 3 min followed by 19 cycles of 98°C for 20 s, 65°C for 30 s, and 72°C for 30 s, with a final extension of 5 min at 72°C. PCR products were purified with 1x AMPure XP beads (Agencourt) and quantified by Qubit. The pooled library contained three samples at 6 pM with 9% PhiX and was sequenced with a 50-cycle single-end MiSeq reagent kit (Illumina). The sequencing reads were aligned to the reference genome (NC$_000913.3$) using the BWA (Burrows-Wheeler Aligner) method. Mapped reads were binned in to 10 kb non overlapping bins using a custom perl script and the number and frequency of insertions in each bin plotted against the chromosome coordinates. The reads considered were filtered for map quality greater than 20 from the same file.

Whole genome sequencing to measure gene dosage. For genomic DNA extraction, the overnight cultures were inoculated in 50 ml of fresh LB media to bring the initial OD of the culture to 0.03 and the flasks were incubated at 37°C with shaking at 200 rpm. Cells were harvested at the maximum growth rate and genomic DNA was isolated using SIGMA GenElute^{TM} Bacterial Genomic DNA Kit using the manufacturer's protocol. Library preparation was carried out using the Truseq Nano DNA low throughput Library preparation kit and Paired end sequencing of genomic DNA was

performed using Illumina Hiseq 2500 platform. The sequencing reads were aligned and mapped to the reference genome (NC_000913.3) using Burrows Wheeler Aligner (BWA) specifying alignment quality and mapping quality thresholds as 20. Read coverage across the genome was calculated for non-overlapping windows of 200 nt each using customized perl scripts.

Whole genome sequencing and analysis of single clones isolated from the FACS populations. Genomic DNA of single clones isolated from sorted populations (Qiagen DNA purification kit) were subjected for paired-end sequencing (2X75) using Illumina Miseq platform. DNA libraries were prepared using the TruSeq Nano DNA LT kit protocol. The sequencing reads were mapped with the reference genome (NC _000913.3) as well as with the known cloned insertion sequence using the BWA (Burrows-Wheeler Aligner) method. The positions of the insertions were identified in each clone using the mate pairing approach for paired-end reads in which the cloned insert sequence is mapped to the read where the genome coordinate maps to the pair.

Nanopore sequencing to confirm the transposon insertion in a rRNA operon. A total of 32 samples were shown to have the insertion in a rRNA operon from whole genome sequencing data. Five of these were subjected to nanopore sequencing using Oxford nanopore technologies. Reads were assembled using a *de novo* assembler (3) to obtain a single contig($\sim$4.6 Mb) for each sample. A BLASTN search was carried out using the cloned insert sequence as a query to confirm the chromosomal position of insertion.

Data availability. The sequencing data are available on the Sequence Read Archive (SRA) (`https://www.ncbi.nlm.nih.gov/sra`) under Bioproject accession IDs PRJNA575574 (WGS Data) and PRJNA575567 (TRADIS data). The (processed) cloned insert FASTA file is available as Supplementary File SF2.

Plate reader analysis.

The growth media used in this study were a "slow growth" medium, M9 minimal medium + 0.5% glucose and "fast growth" medium, M9 minimal medium + 0.5% glucose + 0.2% casaminoacids. All the strains were grown overnight in M9 medium + 0.5% glucose + 0.2% casaminoacids. The next day, they were diluted to 1:1000 for the slow growth medium and 1:10000 for the fast growth medium in a flat bottom 96-well plate. The final volume in each of the culture well was 150 μl. 70 μl of mineral oil (Sigma) was added on top to reduce evaporation of the samples. The plates were incubated at 37°C shaking in the plate reader (Tecan). The optical density (OD_{610}) and the GFP fluorescence at 535 nm were measured every 5 minutes over 21 hours. As a control strain, BW25113 was used, not containing the GFP reporter insertion in the chromosome.

Data analysis of the plate reader experiments

Growth rate and GFP concentration for all the strains were obtained from the data obtained in the plate reader on the optical density at 610 nm (OD) and the fluorescence (GFP) as a function of time. The exponential growth phase is defined as the period during which the OD increases exponentially between two user-defined OD thresholds. The growth rate in exponential phase is defined as the

slope from the linear fit of the log(OD) vs time divided by log(2). The GFP concentration is obtained from the slope of the linear fit of the plot of GFP versus OD.

Measurement of the noise in gene expression

30 to 32 colonies from each population (except for the very low expressing population from round 2, 48 colonies, and round 3 of FACS, 46 colonies) and the parental population of 94 colonies (a total of 658, 32 colonies from 12 different population, 30 from 6 different populations, 48 and 46 from two different populations, 94 form the parental population) were selected randomly from the agar plates. The design for most of the plates was 32 colonies from two different populations and 30 colonies from a third population except for two plates, where we used 48 and 46 wells from one population and 94 form the same population. In total, we had 32 + 32 + 30 = 94 wells for each of the 5 experiments + 1 internal control + 1 BW25113, with a 6th experiment using 48 + 46 wells from sorted populations + 2 controls and a 7th one of 94 wells + 2 controls, yielding 658 colonies. After overnight primary growth in M9 minimal medium with 0.5% Glucose and 0.2% casaminoacids, 1% of the primary culture was diluted to 1:500 and grown in a secondary culture in the same medium until the OD reached 0.5 to 0.7 (mid-log Phase). When the cultures reached mid-log phase, we performed FACS analysis to measure the distribution of fluorescence. 50,000 events were collected for each clone. The first 1000 events were omitted from further analysis. Using the FlowJo software, a kernel density estimate was used to select the contour with the highest density of cells and the center region of the contour was taken for analysis. The magnetic gate plug-in from FlowJo was used to selectively encompass 10,000 events, and the gate was re-adjusted if there was a shift, so that only the most dense centered region events (10,000) were collected for further analysis. The same gate was used for all the clones in a 96-well experiment. A custom-made MATLAB code was used to measure the coefficient of variation (CV), Fano factor, and noise of each sample.

Time-lapse, single cell imaging of the clones and data analysis

Individual colonies were grown overnight in minimal medium containing 0.5% Glucose + 0.1% CAA. The overnight culture was diluted to 1:200 in a secondary culture in the same growth medium. The culture was grown until the OD reached 0.1.

Agar-pad Protocol. Five microliters of culture were added to the center of a 1.25% (w/v) low-melting agar pad made with the same growth medium as the culture. The pad was allowed to dry for half an hour at room temperature and then it was inverted into a small glass dish to be observed under the microscope.

Microscope. A Nikon Inverted microscope ECLIPSE Ti-E with a 100X oil objective was used to perform the live imaging. To correct the drift in focus, we used a Nikon Perfect Focus System. A custom-made temperature control system was used to keep the temperature constant at 30°C throughout the imaging session. The automated x-y stage was used to capture different regions of interest in the agar pad. Time-lapse movies were captured over different positions at intervals of 5 minutes.

Exposure time and Acquisition parameters. For the repressed or silent colonies, the settings were following: exposure time: 200ms, multiplier: 100, lamp 20%. For the low noise colonies, the settings were following: exposure time: 40ms, multiplier: 10, lamp 15%. The reason for the difference is that the signal from low-noise colonies saturated with the settings of high-noise ones, whose signal can be very low.

Data analysis. The contours of the cells were drawn manually in every frame using the ImageJ software. Total GFP intensity was measured for every cell in the lineage tree. The total intensity was divided by the area to obtain the mean fluorescence intensity of each cell in the frame. The background intensity was subtracted to obtain the normalised fluorescence intensity of the cells.

Data sets from the literature

The RegulonDB database (`http://regulondb.ccg.unam.mx/`) was used to extract gene positions. The lists of genes tested for insertion enrichment were obtained from the NuST database (4), which collects data from many published studies related to nucleoid organization(4). The complete list of data sets is presented in Supplementary Table 1.

Each data set has the form of a gene list. The data sets were organized by the type of biological data and the experimental techniques, and divided into the following wide categories: "Genes linked to specific phenotypes"; "Genes next to binding sites of a regulator from binding data"; "Genes sensitive to nucleoid perturbations from transcriptomics experiments"; "Genes with specific annotations"; "Mutated genes in driven evolution experiments"; "Target genes of a regulator". A description of the gene lists used in the analysis is provided in Supplementary Table 1.

The OriC site is the origin of replication of the *E. coli* chromosome. For historical reasons, the coordinates of the *E. coli* circular chromosome do not start at the origin of replication, but at the origin of transfer during conjugation. We set the OriC position at [3,925,744 - 3,925,975] (`https://ecocyc.org/`)

Extended Methods

Estimate of the expected gene-dosage effect in transposition.

This section discusses the model used to understand the null trend in the insertion frequency coming from gene dosage (5; 6). The samples are grown in rich medium and when they are exposed to the transposon, there are a higher number of copies of the chromosome close to the origin than close to the terminus (Figure 1 in the main text).

We proceed to estimate the dosage theoretically. We assume that the $C + D$ periods last 40 + 20 minutes, since we are dealing with fast growth in LB medium, and that the doubling time τ is around 25 minutes (which is the most conservative estimate in terms of dosage bias). Since at time $C + D$ (≈ 60 minutes) the cell divides, a time lag B before initiation is necessary to make the total replication time $B + C + D$ an integer multiple of the doubling time τ, "synchronizing" DNA replication and cell division. Thus, defining $n = \mathrm{Int}\dfrac{C+D}{\tau}$ as the integer number of times that τ divides $C + D$, the following relation has to be satisfied,

$$B + C + D = (n + 1)\tau \ . \tag{S1}$$

More generally (6), we can consider a gene at a chromosomal position defined by its normalized distance from the replicaiton origin (Ori), i.e. $x = 0$ represents a gene in the Ori locus and $x = 1$ a gene in the replication terminus (Ter). The copy number of this gene, $g(x, t)$, depends on its distance from Ori, and changes with time t from the beginning of the cell cycle following

$$g(x,t) := \begin{cases} 2^{n'} & \text{if } 0 < t < (n'+1)\tau - (c(1-x)+D) \\ 2^{n'+1} & \text{if } (n'+1)\tau - (c(1-x)+D) < t < \tau \end{cases} , \tag{S2}$$

where $n' = \mathrm{Int}\left[\dfrac{C(1-x)+D}{\tau}\right]$ is the local number of active replication processes, which depends on the locus position x.

To evaluate the average gene dosage in a cell population of asynchronous cells with an exponential growth we have to consider the distribution of the time into the cell cycle across the population (7). For ideal balanced exponential growth with growth rate μ this distribution is given by $a(t, \mu) = 2\ln(2)\mu 2^{-\mu t}$. Averaging $g(x, t)$ over this distribution, the population-average gene copy number per cell becomes

$$g(x) = \langle g(x,t) \rangle_{population} = \int_0^{\tau} a(t,\mu) g(x,t) dt = 2^{\mu[C(1-x)+D]}. \tag{S3}$$

In order to estimate the mean dosage of inserted promoters, we also have to introduce a model for insertion kinetics. The simplest model relies on the assumption of constant insertion rate. We hypothesize that during a time interval T a cell population that grows exponentially with division time $\langle \tau \rangle$ is exposed to a transposon which inserts its sequence at a constant rate r per time and genome coordinate unit. The expected number of insertions under this assumption can be obtained by the cumulative distribution function. The mean copy number of each coordinate in a cell population is $g(x)$ and we can assume that there is a Poisson process with the same rate (S) in each genome position, so that the dependency of the rate from position x is only due to dosage, $r(x) = g(x)S$. In this case, the estimate for the insertion probability is

$$P(x) = 1 - e^{r(x)T} \simeq r(x)T \simeq g(x)ST = Ag(x) \ , \tag{S4}$$

where we have linearized the expression for small T and $A = ST$ is the only free parameter, which depends on the parameters average gene dosage $g(x)$, time interval T and growth rate μ, all derived from experimental data. The trend of normalized position and the dosage estimate for insertions is shown in Fig. 1B in the main text. This plot shows that this estimate does not correspond well quantitatively to the experimental coverage data.

We verified that a model with time-dependent insertion rate, i.e. where the insertion rate r *increases* with time t, $r(x,t)$, can fit the data, using insertion rate growing as a power law in time. However, there is no empirical motivation to assume such cooperative behavior in insertions that occur *in different cells*.

We now address the simple point that the frequency of an insertion is not biased by population growth, as long as it does not confer a sufficiently strong positive or negative fitness effect. Let f_i be the fraction of insertions in site i and f_j the fraction of insertions in site j. If N is the total number of insertions we will have, at time τ, $Nf_ie^{\alpha\tau}$ insertions in site i and $Nf_je^{\alpha\tau}$ insertions in site j. Then, the total number of insertions will be $Ne^{\alpha\tau}$.

Let $f_{i,j}(\tau)$ be the ratio of the numbers of insertions in site i and j. Then,

$$f_{i,j}(\tau) = \frac{Nf_{i,j}e^{\alpha\tau}}{Ne^{\alpha\tau}} = f_{i,j} \ . \tag{S5}$$

Since $f_{i,j}(\tau) = f_{i,j}$, for all those insertions not affecting the growth rate, exponential propagation of insertions in progeny does not create a bias in the population. If instead an insertion significantly affects the growth rate of the corresponding clone, its presence will be biased in the population.

Null model for insertion enrichment.

This section describes the null model used to score enrichment of insertions in particular regions defined by gene lists. Since the theoretical prediction scores poorly (and even a fit with a time-dependent insertion rate model does not capture quantitatively the shape of the dosage peak around the origin), in the analysis for scoring overlap between insertions and gene lists we decided to use a more conservative way to subtract the dosage, i.e. directly to consider a sliding-window average of the data as background insertion probability. Specifically, we used a window size of 3000 bp, which is the smallest window size that gives a smooth curve with few local features.

In order to investigate statistical tendencies for transposon insertions to be associated to specific chromosomal contexts, we need a suitable null model. This is simple to define using gene lists, as randomizations of the empirical lists having fixed number of genes. In particular, for each tested gene list we simulate 5000 different stochastic realizations.

The definition of enrichment needs a quantity representative of the tendency of finding the insertion fixed in specific chromosomal contexts. We evaluate a discrete integral considering the coverage corresponding to a portion of genome defined by the genes of the list. We call this quantity "coverage integral". It corresponds to the total overlap of the coverage with the genes of a specific list.

We have evaluated the Z-score as significance score for over/under-representation of the overlap between coverage and target gene lists. The Z-score is defined as $Z = \dfrac{X-\mu}{\sigma}$ where X is the empirical value and μ and σ are the mean and the variance relative to a particular sample.

DNA primers used:

To verify the insert on the Tn5 construction vector:
Forward Primer : AATTCTACGAGCGTCGTCGCAGACATGATC
Reverse Primer : GGTCTCAGATGGTAGACGCAGGAAGAGACGAGACAG

Primers used for transposon sequencing experiments (* indicates a phosphorothioate group:
Index primer: AAGAGCGGTTCAGCAGGAATGCCGAGACCGATCTC

3' Tn specific primer:
AATGATACGGCGACCACCGAGATCTACACCCAATATGCGAGAACACCCGAGAAAATTCATCG

3' Sequencing primer: CCCGAGAAAATTCATCGATGATGGTTGAGATGTGTA

Illumina Read 1: ACACTCTTTCCCTACACGACGCTCTTCCGATCT
Illumina Read 2: CGGTCTCGGCATTCCTGCTGAACCGCTCTTCCGATCT

QPCR2.1 : CAAGCAGAAGACGGCATACGA
qPCR2.2 : AATGATACGGCGACCACCGAG

Adapters:
SplA5_top G*AGATCGGTCTCGGCATTCCTGCTGAACCGCTCTTCCGATC*T
SplA5_bottom /5Phos/G*ATCGGAAGAGCGGTTCAGCAGGttttttttttcaaaaaaa*a
SplAP5.2 C*AAGCAGAAGACGGCATACGAGATAAACATCGGAGATCGGTCTCGGCATTC*C
SplAP5.5 C*AAGCAGAAGACGGCATACGAGATACCACTGTGAGATCGGTCTCGGCATTC*C
SplAP5.6 C*AAGCAGAAGACGGCATACGAGATACATTGGCGAGATCGGTCTCGGCATTC*C
SplAP5.12 C*AAGCAGAAGACGGCATACGAGATAGTACAAGGAGATCGGTCTCGGCATTC*C

References

[1] Dennis,P.P., Ehrenberg,M., and Bremer,H. (December, 2004) Control of rRNA synthesis in Escherichia coli: a systems biology approach.. *Microbiology and molecular biology reviews : MMBR,* **68**, 639–668.

[2] Barquist,L., Mayho,M., Cummins,C., Cain,A.K., Boinett,C.J., Page,A.J., Langridge,G.C., Quail,M.A., Keane,J.A., and Parkhill,J. (April, 2016) The TraDIS toolkit: sequencing and analysis for dense transposon mutant libraries.. *Bioinformatics (Oxford, England),* **32**, 1109–1111.

[3] Koren,S., Walenz,B.P., Berlin,K., Miller,J.R., Bergman,N.H., and Phillippy,A.M. (May, 2017) Canu: scalable and accurate long-read assembly via adaptive , javax.xml.bind.JAXBElement@21ca03c5, -mer weighting and repeat separation.. *Genome research,* **27**, 722–736.

[4] Scolari,V.F., Zarei,M., Osella,M., and Cosentino Lagomarsino,M. (2012) NuST: analysis of the interplay between nucleoid organization and gene expression. *Bioinformatics,* **28**(12), 1643–1644.

[5] Cooper,S. and Helmstetter,C.E. (February, 1968) Chromosome replication and the division cycle of Escherichia coli B/r.. *Journal of molecular biology,* **31**, 519–540.

[6] Osella,M. and Cosentino Lagomarsino,M. (January, 2013) Growth-rate-dependent dynamics of a bacterial genetic oscillator.. *Physical review. E, Statistical, nonlinear, and soft matter physics,* **87**, 012726.

[7] Marathe,R., Bierbaum,V., Gomez,D., and Klumpp,S. (07, 2012) Deterministic and Stochastic Descriptions of Gene Expression Dynamics. *Journal of Statistical Physics,* **148**.

[8] Kahramanoglou,C., Seshasayee,A.S.N., Prieto,A.I., Ibberson,D., Schmidt,S., Zimmermann,J., Benes,V., Fraser,G.M., and Luscombe,N.M. (Mar, 2011) Direct and indirect effects of H-NS and Fis on global gene expression control in Escherichia coli.. *Nucleic Acids Res,* **39**(6), 2073–2091.

Supplementary Figures

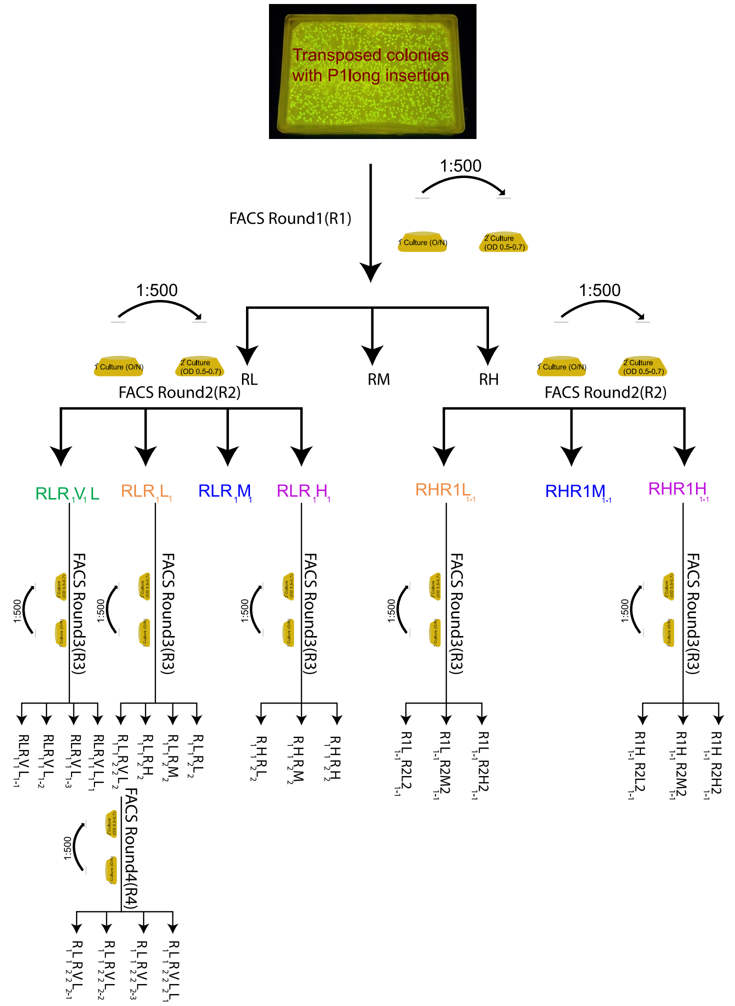

Figure S1: **Full illustration of the sorting pipeline starting from the parental population and the nomenclature used in our experiment.** The parental population (the cells that underwent transposition) was grown overnight in M9 medium with 0.5% Glucose + 0.2% CAA ("fast growth" medium) and was diluted to 1:500 to grow in secondary culture with the same medium the following day until the OD reached 0.5 to 0.7. These cultures underwent first round of FACS to yield three population by the level of GFP expression (Low (RL), Medium (RM) and High (RH)). Subsequently the Low (RL) and High (RH) underwent the second round of FACS, the former (RL) yielded 4 population, RLR1V1L1 (Very low), RLR1L1 (Low), RLR1M1 (Medium) and RLR1H1 (High) while the latter (RH) yielded three population, RHR1L1-1 (Low), RHR1M1-1 (Medium) and RHR1H1-1 (High). From the second round of FACS, three populations, RLR1V1L1 (Very low), RLR1L1 (Low), and RLR1H1 (High) were selected from the low expressing population while 2 populations, RHR1L1-1 (Low) and RHR1H1-1 (High) from the high expressing population were selected for the third round of FACS. RLR1V1L1 (Very low) gave rise to 4 different populations, RLR1V1L1L1, RLR1V1L1-1, RLR1V1L1-2, RLR1V1L1-3 and RLR1L1 (Low) also gave rise to 4 different population, R1L1R2V2L2, R1L1R2L2, R1L1R2M2, R1L1R2H2 while RLR1H1 (High) gave rise to 3 different populations, R1H1R2L2, R1H1R2M2 and R1H1R2H2. From the high expressing populations, RHR1L1-1 and RHR1H1-1 gave rise to R1L1-1R2L21-1, R1L1-1R2M21-1, R1L1-1R2H21-1, and R1H1-1R2L21-1, R1H1-1R2M21-1, R1H1-1R2H21-1, respectively. Only the very low expressing population, R1L1R2V2L2 from the third round of FACS gave rise to 4 different very low expressing populations, R1L1R2V2L2-1, R1L1R2V2L2-2, R1L1R2V2L2-3, R1L1R2V2L2-4.

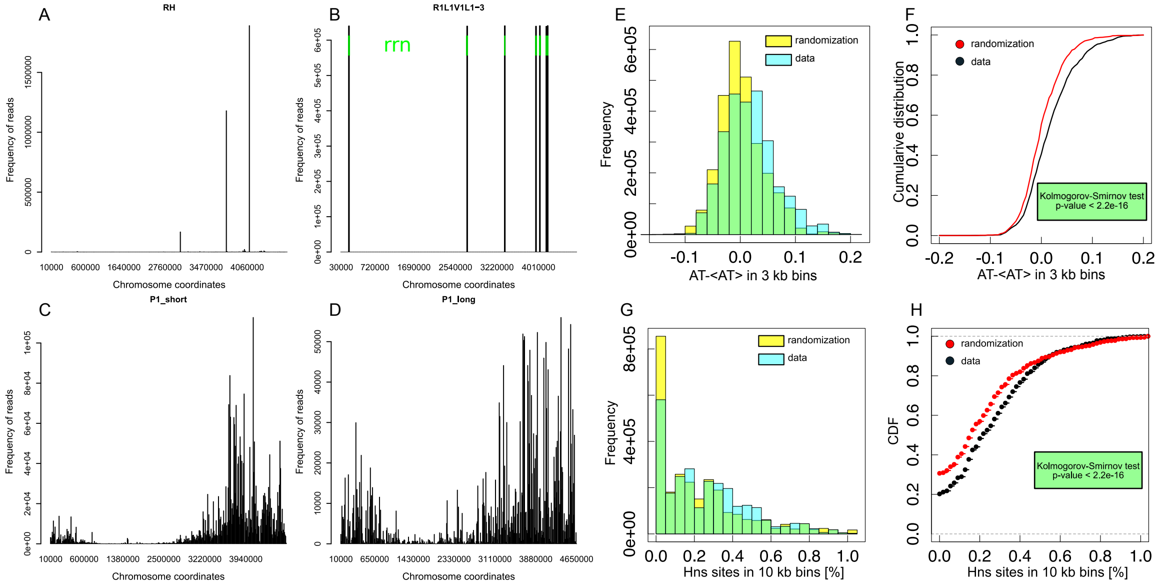

Figure S2: **Insertion frequency plots and AT bias.** A): Highly expressing sorted (RH) population shown in Figure 1D shown in linear scale. B): Insertions in very low expressing sorted population (RLR1V1L1), shown in linear scale, transposon insertions in rRNA operons are visible close to the origin. C) and D): Insertion frequency plots for parental populations of P1-long and P1-short promoters respectively. The plots were generated from TraDis Data by binning the mapped position of the each read into 10kb non overlapping bins, and the frequency of insertions of each bin was plotted against the chromosome coordinate. E) and F) Insertions have a significant positive bias in AT-richness compared to the background. E): Histograms of deviations from average in AT-richness $(\%AT - < \%AT >)$ in the sequences surrounding insertions from the experimental data and from a randomized sample of the background sequences of the genome. $\%AT$ corresponds to a local average in 3Kb intervals around each insertion; $< \%AT >$ refers to the average on the entire genome. F): comparison of the cumulative distributions and results of the Kolmogorov-Smirnov test (in both cases the p-value is smaller than the smallest represented by the ks.test R script). G) and H) Insertions have a positive bias for H-NS binding. The plots report the distribution of the fraction of the 10kb region around an insertion covered by H-NS binding sites (ChIPseq data from ref. (8)), compared to the same quantity for randomized insertion sites.

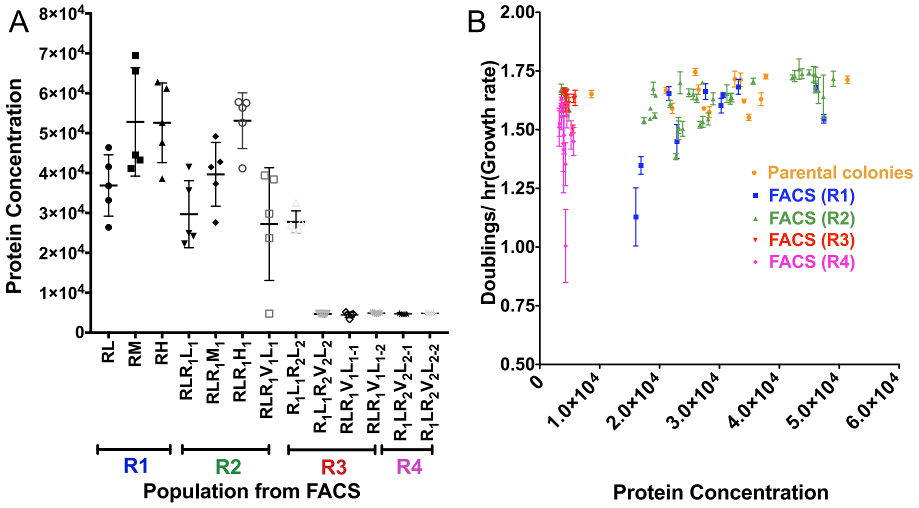

Figure S3: **Expression levels of clones from sorted populations measured by fluorimetry, and growth rate.** (A) The fluorimeter expression data are in agreement with the FACS population histograms. GFP expression (total fluorescence over OD) of 5 individual strains randomly chosen from each of the different rounds of FACS sorted population. All the strains were grown in rich medium (0.5% Glucose + 0.2% CAA). Error bars represent standard error of the mean (SEM). R1, R2, R3, R4 denote the first, second, third and fourth round of FACS. Expression is low for very low expressing population (VL in R3 and in R4) and significantly different from the other sorted populations ($P < 0.001$, One-way ANOVA, Tukey's multiple comparison test comparing R1, R2 with R3 and R4). The fluorimeter expression data are in agreement with the FACS population histograms. (B) Scatter plot of expression levels and population growth rates for clones from the different rounds.

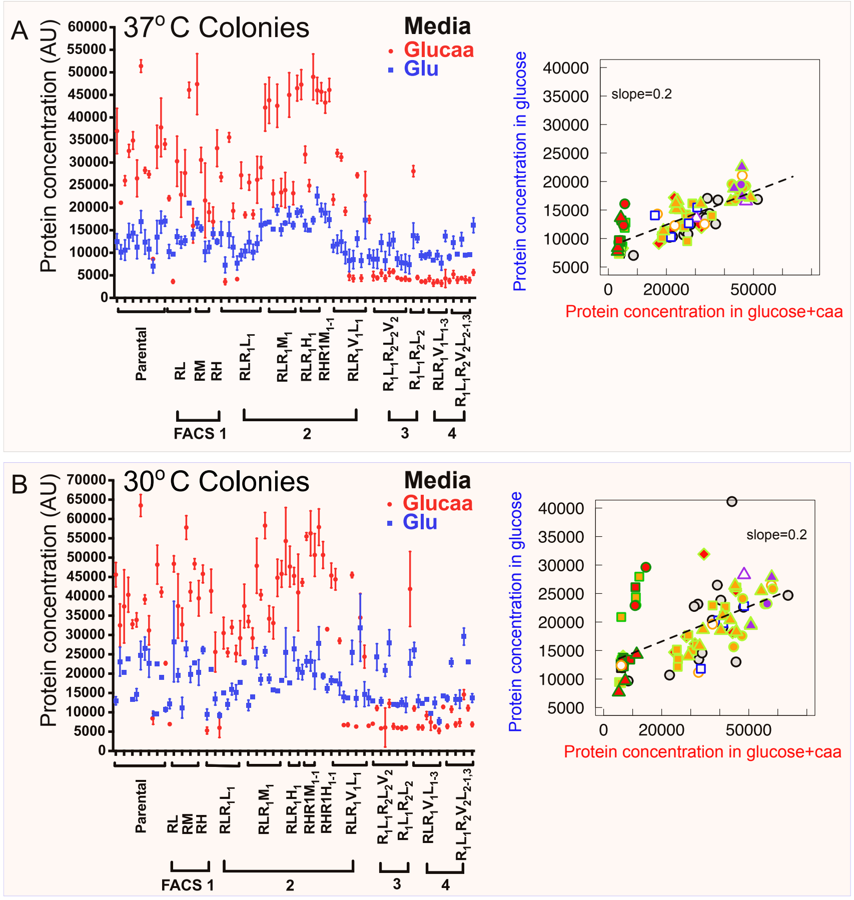

Figure S4: **Insertions at the ribosomal regions are highly repressed in different growth conditions.** The expression levels of these colonies are lower when they are grown in a fast-growth medium (M9 + 0.5% Glucose + 0.2% CAA) compared to the slower-growth medium (M9 + 0.5% Glucose) irrespective of the temperature grown (37°C and 30°C). Panel A) reports the gene expression of 90 clones from different rounds of FACS grown in different media (fast growth, Glucose + CAA, compared to the slow growth medium, Glucose at 37°C). Error bars represents standard deviation (SD), n = 3. Panel B) represents the comparison of colonies grown in different media (fast vs slow growth medium) at 30°C, error bars represent SD, n = 2. The scatter plots on the right side of each panel compare gene expression of the same clone in the two growth conditions (color/symbol codes defined in Supplementary Fig. 5). Note that while most of the other clones increase in expression in the faster growth medium (in agreement with a ribosomal promoter) the very-low expression (VL) ones decrease with the faster growth rate, in agreement with the idea that their expression is decreased by the activity of a ribosomal promoter. The comparison between the two temperatures shows that there is a subset of clones that increase in expression in the slower growth Glucose medium at low temperature, spanning different sorted populations. Note that thereare two growth rate dependence results, the first is the fact that we grew the cells in two growth media and we observed an increase in gene expression in the richer medium, as expected from the rrnBP1 promoter, the second is that within a given growth medium different clones grew at different rates (Fig. S3B).

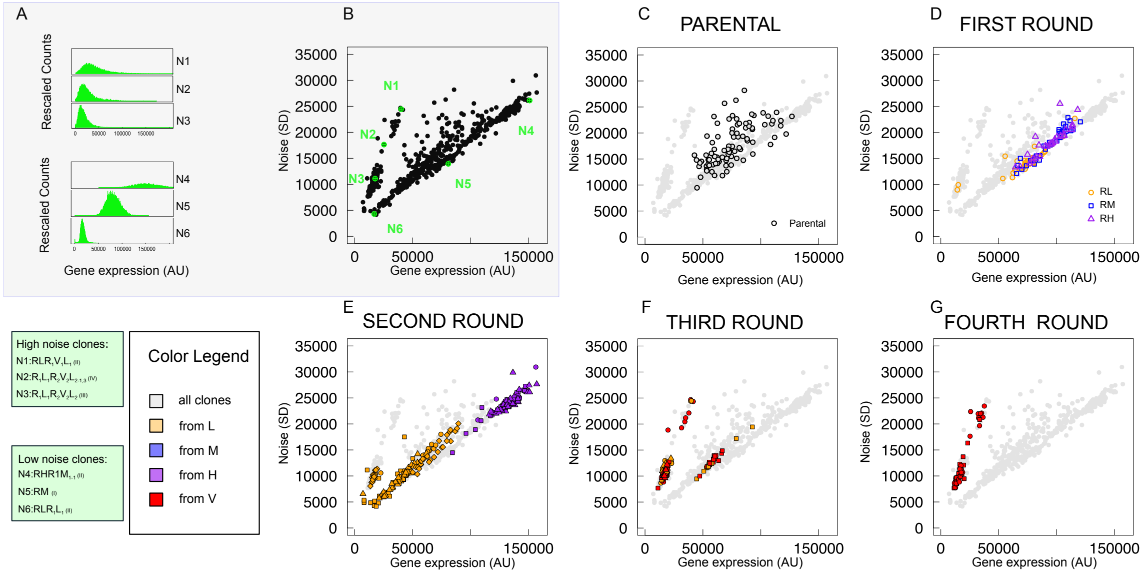

Figure S5: **Gene expression levels and noise of clonal populations from FACS.** The figure recapitulates the main results of the selected clonal populations (hence carrying the same insertions). A) and B) N1-N6 are six representative clones chosen from all the measured ones. Panel B (the same plot shown in Figure 3A) shows their gene expression mean level and noise (SD), while panel A compares the histograms of their gene expresion levels. Panels C-G use the same plot to show the noise properties of the clones coming from different rounds of FACS sorting.

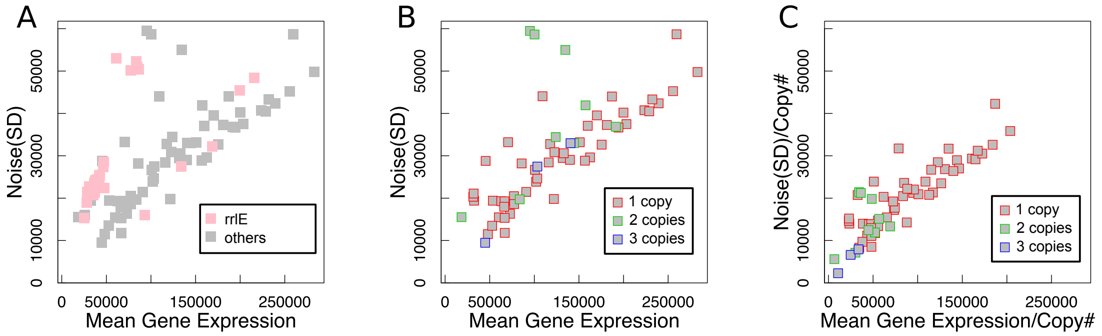

Figure S6: **Gene expression noise categories of sequenced clones with varying insertion copy number.** A) Mean vs SD plots of rrlE vs non-rrlE insertions. Noisy promoters mostly associate with rrlE insertions in sequenced clones. B) Mean vs SD plots of non-rrlE sequenced clonal populations (a subset of the data in Supplementary Fig. S5), where the points are colored by number of insertions. C) Different plot of the same data, where gene expression normalized per copy number. Removal of the effect of copy number fold change in the expression levels reconducts many non-rrlE outliers to the cluster of low-noise promoters.

Supplementary Tables

Genes linked to specific phenotypes (essential genes and genes responsive to external conditions)									Mutated genes		
Multi-stress responsive genes (from Nichols et al 2011)	Conditionally essential genes due to auxotrophy (from Nichols et al 2011)	Conditionally essential genes in rich media (from Nichols et al 2011)	Genes whose expression is affected by growth conditions (from Regulon DB)	Genes whose expression is affected by the carbon source (from RegulonDB)	List of E. coli essential genes (from Baba et al 2006)	List of E. coli essential genes (from Gerdes et al 2003)			List of mutated genes in E. coli long-term evolution experiment through 20,000 generations (Barrick et al 2009)	List of mutated genes in E. coli long-term evolution experiment through 40,000 generations (Barrick et al 2009)	
Genes next to binding sites of a regulator from binding data (ChIP-chip, ChIP-seq, etc)											
Putative RNA-polymerase target genes during rapid growth from ChIP-chip experiments (from Grainger et al 2005)	Genes overlapping with heEPODs (highly expressed extended protein occupancy domains) (from Vora et al 2009)	Genes overlapping with tsEPODs (transcriptionally silent extended protein occupancy domains) (from Vora et al 2009)	Putative CRP target genes from ChIP-chip experiments (from Grainger et al 2005)	Putative FIS target genes from ChIP-chip experiments (from Cho et al 2006)	Putative FIS target genes from ChIP-seq experiments (Kahramanoglu et al 2010)	Putative FIS target genes in early-exponential phase from ChIP-seq experiments (from Kahramanoglu et al 2010)	Putative FIS target genes in mid-exponential phase from ChIP-seq experiments (from Kahramanoglu et al 2010)	Putative FIS target genes in mid-log phase from ChIP-chip experiments (from Grainger et al 2006)	Putative FNR target genes in stationary phase from ChIP-chip experiments (from Grainger et al 2007)	Putative FNR target genes in midlog phase in the presence of oxygen from ChIP-chip experiments (from Grainger et al 2007)	Putative FNR target genes in midlog phase from ChIP-chip experiments (Grainger et al. 2007)
Putative H-NS target genes from ChIP-chip experiments (from Oshima et al 2006)	Putative H-NS target genes from ChIP-seq experiments (Kahramanoglu et al 2010)	Putative H-NS target genes in early-exponential phase from ChIP-seq experiments (from Kahramanoglu et al 2010)	Putative H-NS target genes in mid-exponential phase from ChIP-seq experiments (from Kahramanoglu et al 2010)	Putative H-NS target genes in mid-log phase from ChIP-chip experiments (from Grainger et al 2006)	Putative H-NS target genes in stationary phase from ChIP-seq experiments (from Kahramanoglu et al 2010)	Putative H-NS target genes in transition-to-stationary phase from ChIP-seq experiments (from Kahramanoglu et al 2010)	Putative IHF target genes in mid-log phase from ChIP-chip experiments (from Grainger et al 2006)				
Genes sensitive to nucleoid perturbations from transcriptomics experiments											
FIS knockout sensitive genes in all stages of growth (from Bradley et al 2007)	FIS knockout sensitive genes in the early-exponential growth phase (from Bradley et al 2007)	FIS knockout sensitive genes in the late-exponential growth phase (from Bradley et al 2007)	FIS knockout sensitive genes in the mid-exponential growth phase (from Bradley et al 2007)	FIS knockout sensitive genes in the stationary phase (from Bradley et al 2007)	FIS-knockout sensitive genes under negative-supercoiling (from Blot et al 2006)	FIS-knockout sensitive genes under positive-supercoiling (from Blot et al 2006)	H-NS knockout sensitive genes under negative-supercoiling (from Blot et al 2006)	H-NS knockout sensitive genes under positive-supercoiling (from Blot et al 2006)	Supercoiling sensitive genes under FIS knockout (from Blot et al 2006)	Supercoiling sensitive genes (from Blot et al 2006)	Supercoiling sensitive genes under H-NS knockout (from Blot et al 2006)
Genes with specific annotations				Target genes of a regulator (according to external databases)							
Putative horizontally transferred genes (from HGT-DB)	Genes related to small RNAs (from Regulon DB)	Genes whose protein products have transmembrane domains (from Ensembl Bacteria)	Genes with paralogs (from Ensembl Bacteria)	Genes regulated by small RNAs (from Regulon DB)	Genes regulated by the sigma factor 24 (from Regulon DB)	Genes regulated by the sigma factor 28 (from Regulon DB)	Genes regulated by the sigma factor 32 (from Regulon DB)	Genes regulated by the sigma factor 38 (from Regulon DB)	Genes regulated by the sigma factor 54 (from Regulon DB)	Genes regulated by the sigma factor 70 (from Regulon DB)	

Table S1: **Gene lists used in the enrichment analysis, grouped by category.** Lists were divided according to the types of biological data and the experimental techniques. See ref. (4) for details. The symbols are the ones used in Fig. 2 in the main text.

GENE SET	Z-SCORE (p1 long)	Z-SCORE (p1 short)	Z-SCORE (p1 short)
Putative H-NS target genes from ChIP-seq experiments (Kahramanoglou et al 2010)	19,7	27.3	27,3
Putative H-NS target genes from ChIP-chip experiments (from Oshima et al 2006)	13,9	17,1	17,1
Putative H-NS target genes in early-exponential phase from ChIP-seq experiments (from Kahramanoglou et al 2010)	10,5	15,5	15,7
Genes overlapping with tsEPODs (transcriptionally silent extended protein occupancy domains) (from Vora et al 2009)	9,5	13,5	13,6
Putative H-NS target genes in mid-exponential phase from ChIP-seq experiments (from Kahramanoglou et al 2010)	9,2	13,4	13
Putative H-NS target genes in transition-to-stationary phase from ChIP-seq experiments (from Kahramanoglou et al 2010)	7,9	11,4	11,5
FNR target genes in midlog phase in the presence of oxygen from ChIP-chip experiments (from Grainger et al 2007)	6,7	7,2	7,7
Putative H-NS target genes in stationary phase from ChIP-seq experiments (from Kahramanoglou et al 2010)	6,6	10,8	10
Putative FNR target genes in midlog phase from ChIP-chip experiments (Grainger et al. 2007)	6,5	8,9	9
Putative FNR target genes in midlog phase in the presence of oxygen from ChIP-chip experiments (from Grainger et al 2007)	6,4	7,2	7,2
Putative horizontally transferred genes (from HGT-DB)	5,3	4,1	4,1
Putative FIS target genes from ChIP-seq experiments (Kahramanoglou et al 2010)	3,8	7,1	7
H-NS knockout sensitive genes under negative-supercoiling (from Blot et al 2006)	3,7	6,6	6,7

Table S2: **Groups of genes regarding HNS or horizontally transferred genes result over-represented.** The table reports the gene sets that result over-represented (Z-score $> +5$ in at least one experiment).

GENE SET	Z-SCORE (p1 long)	Z-SCORE (p1 short)	Z-SCORE (p1 short)
List of E. coli essential genes (from Gerdes et al 2003)	-5,2	-6,3	-6,2
List of E. coli essential genes (from Baba et al 2006)	-4,4	-4,5	-4,4
Putative RNA-polymerase target genes during rapid growth from ChIP-chip experiments (from Grainger et al 2005	-3,4	-2,5	-2,7
Supercoiling sensitive genes (from Blot et al 2006)	-2,6	-2	-1,8
Supercoiling sensitive genes under FIS knockout (from Blot et al 2006)	-2,4	-0,3	-0,2
Genes overlapping with heEPODs (highly expressed extended protein occupancy domains) (from Vora et al 2009)	-2,2	-0,4	-0,5

Table S3: **Groups of essential genes result under-represented.** The table reports the gene sets that result under-represented (Z score < -3 in at least one insertion experiment).

GENE SET	Z-SCORE (p1 long)	Z-SCORE (p1 short)	Z-SCORE (p1 short)
Putative FIS target genes in mid-log phase from ChIP-chip experiments (from Grainger et al 2006)	3,8	3,2	3
H-NS knockout sensitive genes under positive-supercoiling(from Blot et al 2006)	3,7	4,1	4,9
Putative IHF target genes in mid-log phase from ChIP-chip experiments (from Grainger et al 2006)	3,6	2	2,1
Putative FIS target genes from ChIP-chip experiments (from Cho et al 2006)	3,5	3,1	2,2
Putative CRP target genes from ChIP-chip experiments (from Grainger et al 2005)	2,4	3,5	3,2

Table S4: **Groups of Fis,IHF,CRP target genes result over-represented considering a lower limit** The table reports the gene sets that result over-represented (Z score > 3 and < 5 in at least one insertion experiment).

	P1 long RL	P1 long RH	P1 short 1	P1 short 2
P1 long	0.86	0.50	0.97	0.97
P1 long RL		0.60	0.83	0.84
P1 long RH			0.51	0.50
P1 short 1				0.99

Table S5: **A correlation exists between Z scores from different strains** Correlation between Z scores from different stains is represented by the Pearson correlation coefficient. P1 long is the parental colony, then we have the first round high expression (RH) and low-expression (RL) sorted populations. Different colors correspond to different correlation levels.

POPULATION	GENE SET	Z-SCORE
RL	Putative H-NS target genes from ChIP-seq experiments (Kahramanoglou et al 2010)	8,4
	Putative H-NS target genes from ChIP-chip experiments (from Oshima et al 2006)	5,8
	Putative H-NS target genes in early-exponential phase from ChIP-seq experiments (from Kahramanoglou et al 2010)	3,8
	Putative horizontally transferred genes (from HGT-DB)	3,7
	Putative H-NS target genes in mid-exponential phase from ChIP-seq experiments (from Kahramanoglou et al 2010)	3,4
	Putative H-NS target genes in transition-to-stationary phase from ChIP-seq experiments (from Kahramanoglou et al 2010)	3,2
	Putative H-NS target genes in stationary phase from ChIP-seq experiments (from Kahramanoglou et al 2010)	2,4
	Genes overlapping with tsEPODs (transcriptionally silent extended protein occupancy domains) (from Vora et al 2009)	2,2
RH	Genes overlapping with heEPODs (highly expressed extended protein occupancy domains) (from Vora et al 2009)	5,4
	Genes overlapping with tsEPODs (transcriptionally silent extended protein occupancy domains) (from Vora et al 2009)	3,4

Table S6: **Only the subpopulation made of low-expression clones is consistent with the result of the whole population.** The data in this table refer to Z-scores for the first round high expression (RH) and low-expression (RL) FACS-sorted populations. The table reports the gene sets that result over-represented with Z-score $> +3$.

Supplementary Files

The Excel supplementary file SF1 *SF1_Suppl_ListColoniesNew.xlsx* contains a list of all the single insertion clonal strains analyzed in detail, including insertion coordinate and measured gene expression parameters (level and noise).

Legend:

Column A: Denotes the population where the clone comes from.

Column B: Describes the origin of the clone and which round of FACS the population came from.

Column C: The ID of the clonal colony (defined as its position in the 96-well plate, arranged column wise).

Column D: The coordinate position of the insertion in the genome, from sequencing. 'N' denotes the positions in the "rrn" regions (note that we do not know the location of exact insertion in very low expressing populations, since they are paired with different rrn operons such as 2728Kb , 4210 kb, 3426kb, 4169kb).

Column E: 'Y' denotes whether each clonal population underwent flow cytometry or not, to measure the CV, Mean and noise of the expression of GFP in each individual strain.

Column F: 'Y' denotes whether the fluorimeter measurements were made for this clone.

Column G: indicates the average protein concentration of individual colonies grown in Glucose at 37 degrees (n=3).

Column H: indicates the average protein concentration of individual colonies grown in Glucose + CAA at 37 degrees (n=3).

Column I: describes the average growth rate (doublings/hour) of individual colonies grown in Glucose at 37 degrees.

Column J: describes the average growth rate (Doublings/hour) of individual colonies grown in Glucose + CAA at 37 degrees.

Column K: describes the mean (Log) fluorescence measurements of individual colonies that underwent flow cytometry.

Column L: describes the CV (Log) fluorescence measurements of individual colonies that underwent flow cytometry.

The supplementary file SF2 *SF2Cloned_Transposon_insert.fa.gz* contains the FASTA file of the cloned insert.
